# Intracellular gold nanoparticles enhance non-invasive radiofrequency thermal destruction of human gastrointestinal cancer cells

**DOI:** 10.1186/1477-3155-6-2

**Published:** 2008-01-30

**Authors:** Christopher J Gannon, Chitta Ranjan Patra, Resham Bhattacharya, Priyabrata Mukherjee, Steven A Curley

**Affiliations:** 1 Department of Surgical Oncology, University of Texas M. D. Anderson Cancer Center, Houston, Texas, USA; 2 The Department of Biomedical Engineering, Mayo Clinic, Rochester, Minnesota, USA

## Abstract

**Background:**

Novel approaches to treat human cancer that are effective with minimal toxicity profiles are needed. We evaluated gold nanoparticles (GNPs) in human hepatocellular and pancreatic cancer cells to determine: 1) absence of intrinsic cytotoxicity of the GNPs and 2) external radiofrequency (RF) field-induced heating of intracellular GNPs to produce thermal destruction of malignant cells. GNPs (5 nm diameter) were added to 2 human cancer cell lines (Panc-1, Hep3B). 3-(4,5-Dimethylthiazol-2-yl)-2,5-diphenyltetrazolium bromide (MTT) assay and propidium iodide-fluorescence associated cell sorting (PI-FACS) assessed cell proliferation and GNP-related cytotoxicity. Other GNP-treated cells were exposed to a 13.56 MHz RF field for 1, 2, or 5 minutes, and then incubated for 24 hours. PI-FACS measured RF-induced cytotoxicity.

**Results:**

GNPs had no impact on cellular proliferation by MTT assay. PI-FACS confirmed that GNPs alone produced no cytotoxicity. A GNP dose-dependent RF-induced cytotoxicity was observed. For Hep3B cells treated with a 67 μM/L dose of GNPs, cytotoxicity at 1, 2 and 5 minutes of RF was 99.0%, 98.5%, and 99.8%. For Panc-1 cells treated at the 67 μM/L dose, cytotoxicity at 1, 2, and 5 minutes of RF was 98.5%, 98.7%, and 96.5%. Lower doses of GNPs were associated with significantly lower rates of RF-induced thermal cytotoxicity for each cell line (P < 0.01). Cells not treated with GNPs but treated with RF for identical time-points had less cytotoxicity (Hep3B: 17.6%, 21%, and 75%; Panc-1: 15.3%, 26.4%, and 39.8%, all P < 0.01).

**Conclusion:**

We demonstrate that GNPs 1) have no intrinsic cytotoxicity or anti-proliferative effects in two human cancer cell lines *in vitro *and 2) GNPs release heat in a focused external RF field. This RF-induced heat release is lethal to cancer cells bearing intracellular GNPs *in vitro*.

## Background

Radiofrequency ablation (RFA) is now used in clinical practice to treat some malignant tumors, yet it suffers from serious limitations [1-4]. These shortcomings include: 1) RFA is currently an invasive treatment requiring insertion of needle electrodes directly into the tumor(s) to be treated; 2) incomplete tumor destruction occurs in 5% – 40% of the treated lesions, particularly if lesions are > 4–5 cm in diameter; 3) the treatment is nonspecific with both malignant and normal tissues around the needle electrode undergoing thermal injury; 4) complications arise in up to 10% of patients, frequently related to thermal injury to normal tissues; 5) and invasive RFA is limited to treatment of tumors in only a few organ sites (liver, kidney, breast, lung, bone) [5,6]. Interestingly, the tissue penetration in humans by focused external RF energy fields is known to be excellent [7]. In theory, non-invasive RF treatment of malignant tumors at any site in the body should be possible, but such a treatment would require the presence of intracellular or intratumoral agents that release heat under the influence of the RF field. For such a novel RF treatment approach to be effective, it will require identification of agents that have little or no intrinsic cellular or tissue toxicity that can also be targeted or directed to malignant cells while sparing normal cells. Clearly, a non-invasive approach with the potential to treat many types of cancers effectively with minimal or no toxic effects to normal cells would be highly beneficial.

Nanoparticles have piqued the interest of the medical community for use in cancer diagnosis, treatment, and as delivery vectors for biologic or pharmacologic agents [8-16]. The ability to affect diagnostic or therapeutic changes on a nanoscale could provide significant gains in medical care. Gold nanoparticles (GNPs) are particularly interesting for several reasons. First, they are easily prepared. Additionally, the binding of molecules to the GNPs in order to target cancer cells, including antibodies, carbohydrates, and pharmacologic agents, is easily achieved [17-21]. Also, the GNPs themselves have anti-angiogenic properties [13].

A previous study revealed that gold-silica nanoshells release significant heat when exposed to near-infrared (NIR) light (650–950 nm) and have been used to produce thermal cytotoxicity *in vitro *[22]. Unfortunately, this treatment approach is mechanistically limited to use in superficial malignant tumors because of the minimal tissue penetration (< 2–3 cm depth) by NIR wavelength light [23]. However, the gold-silica nanoshell study demonstrated that nanogold has potential clinical use as a thermal conductor of non-invasive energy sources. Gold, like most metals, is an excellent conductor of electrical and thermal energy, thus we studied the potential role of GNPs as intracellular molecules that would release heat when treated with RF irradiation.

We hypothesized that 1) the addition of GNPs to hepatocellular and pancreatic human cancer cell lines would not be intrinsically cytotoxic to the cells and 2) cancer cells containing GNPs exposed to a focused, non-invasive RF field would develop lethal, thermal-induced injury.

## Results

### GNP heating

**Figure 1:**
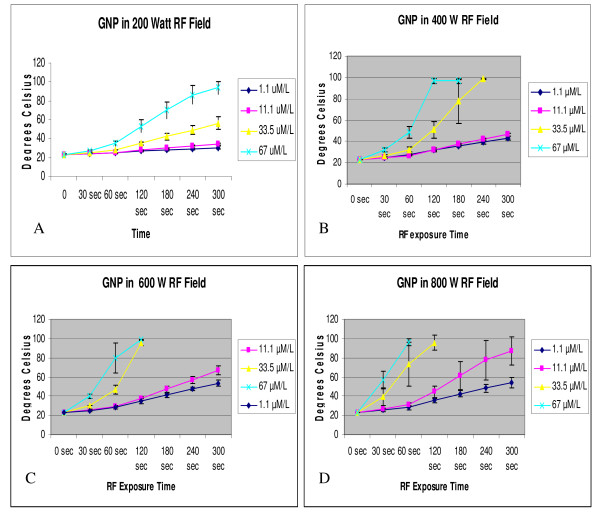
Thermographic results of heating of solutions of gold nanoparticles (GNPs) exposed to external radiofrequency (RF) fields at different RF generator power outputs. Panel A: Graphic depiction of heating rate of deionized water with increasing concentrations of GNPs treated at 200 W of power. B. RF treatment at 400 W of power. C. RF treatment at 600 W of power. D. RF treatment at 800 W of power. Heating curves which conclude prior to 300 seconds are indicative of specimen boiling.

Heating of GNPs with the external RF device occurred in a nonlinear fashion (Fig. 1). Increasing GNP concentration and increasing RF generator power (increasing field voltage) both contributed to increased total heating and rate of heating of water to a boiling point. Figure 1 displays representative heating curves for each concentration of GNPs in deionized water tested at 200 Watts (W), 400 W, 600 W, and 800 W of RF generator power.

### GNP cytotoxicity

Initial evaluation of the GNPs for constitutive anti-proliferative effects against Hep3B and Panc-1 were required before proceeding with RF experimentation. Therefore, MTT assays with serial dilutions of GNPs were performed and revealed no significant effect on Panc-1 or Hep3B cellular proliferation at any of the concentrations measured (1, 10, or 67 μM/L versus media alone). Specifically, Hep3B absorbance as a percentage of untreated controls was 100 ± 5%, 98 ± 6%, and 86 ± 8%, respectively for the GNP concentrations of 1, 10, and 67 μM/L. Similarly, Panc-1 absorbance was negligibly different between concentrations of GNPs and media (98 ± 10%, 90 ± 11%, and 81 ± 9% for 1, 10, and 67 μM/L GNPs). While there is less absorbance at the highest concentration of GNPs (67 μM/L), this absorbance remains within the standard deviation of the DMEM media controls for both Hep3B and Panc-1.

**Figure 2:**
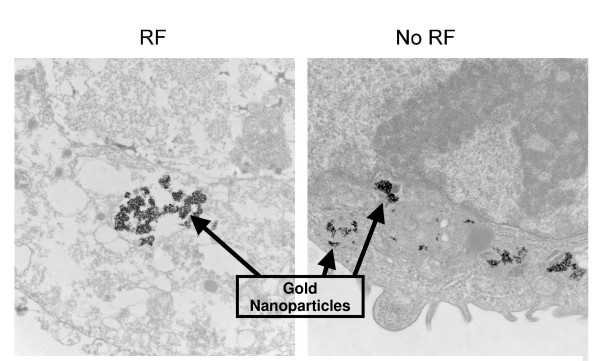
Transmission electron microscopy of Panc-1 cells treated with 67 μM/L gold nanoparticles. Panel 1: 2 minutes of external radiofrequency (RF) field treatment. Note loss of nuclear stability and prominent vacuolization. Panel 2: No RF treatment. Nuclear integrity and normal appearing organelles.

GNPs alone at all concentrations produced no evidence of necrosis in either Hep3B or Panc-1 cells; both cell lines displayed normal cell cycle elements by PI-FACS (data not shown). There was also no major cellular distortion present on TEM images of Panc-1 cells exposed to GNPs alone (Fig. 2, Panel 2). All organelles are intact and all cells imaged are unchanged except for the intracellular presence of GNPs within endosomal structures. This was also true for Hep3B cells; there was no evidence of cellular disruption or organelle damage in the presence of intracellular GNPs (images not shown).

### External RF treatment of cells

Both Hep3B and Panc-1 cells treated with 67 μM/L GNPs and then exposed to the external RF field had markedly higher rates of cell death than the control samples not treated with GNPs at all time-points as measured by PI-FACS (p < 0.01). These results are included in Table 1. Cells treated with external RF after a GNP dose of 1 μM/L had no increased cytotoxicity compared with control cells grown only with media (no GNPs). Cells receiving 10 μM/L GNPs had slightly, but not significantly greater cytotoxicity compared to cells treated without GNPs (data not shown).

**Table 1:** External radiofrequency (RF) field treatment of Panc-1 human pancreatic adenocarcinoma and Hep 3B human hepatocellular cancer cell cultures

	**Cell Type and Treatment**					
RF Exposure	Hep 3B Control Cell Death (%)	Hep 3B GNPs Cell Death (%)	P Value	Panc-1 Control Cell Death (%)	Panc-1 GNPs Cell Death (%)	p Value

**5 minutes**	75.0 ± 12.2	99.8 ± 3.1	0.4	39.8 ± 34.0	96.5 ± 8.4	0.001
**2 minutes**	21 ± 14.1	98.5 ± 0.5	0.001	26.4 ± 15.8	98.7 ± 3.7	0.001
**1 minute**	17.6 ± 8.4	99.0 ± 0.2	0.001	15.3 ± 9.8	98.5 ± 2.1	0.001

GNPs = gold nanoparticles 67 μM/L concentration

**Figure 3:**
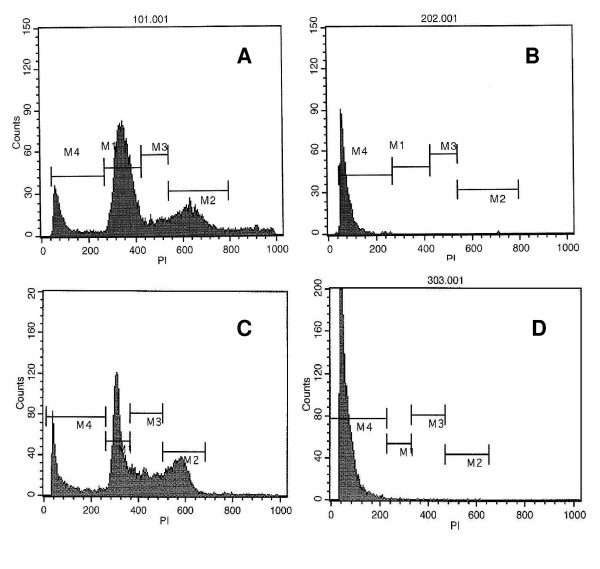
Propidium Iodide-Fluorescent Activated Cell Sorting (PI-FACS) representative graphs. Each sample from one minute radiofrequency (RF) treatment with and without gold nanoparticles (GNPs) at 67 μM/L. Panel A: Hep 3B human hepatocellular cancer cells control with DMEM; Panel B: Hep 3B GNPs; Panel C: Panc-1 human pancreatic cancer cells control with DMEM; Panel D: Panc-1 GNPs.

Shorter exposure times resulted in decreased amounts of cellular death in control samples (< 20% at 1 minute, < 27% at 2 minutes), while the 67 μM/L GNP-treated samples showed on average more than 98% of cells killed at all time-points of RF exposure (both cell lines at both 1 and 2 minutes, Table 1). This killing differential was statistically significant for each time-point when compared to control except for the Hep3B-5 minute sample. The control samples in this 5 minute Hep3B group averaged 75.0% cellular death. The final temperatures recorded for each sample are generally higher for each of the GNP samples tested (Table 1). Representative PI-FACS graphs of cell viability following RF treatment are demonstrated in Figure 3. Interestingly, there was not a significant difference in media temperatures in the RF-treated cells comparing control cells (no GNPs) to cells with various concentrations of GNPs (data not shown). This suggests that heat release from GNPs in the microenvironment of the cells is sufficient to produce lethal injury in the cells even though the concentration of GNPs in the cells is not sufficient to produce significant heating of the relatively large volume media solutions.

## Discussion

The application of nanomaterials to the biohealth arena is an exciting prospect given that most cellular chemical and enzymatic interactions occur on the nanoscale. Therefore, the ability to manage or modify these processes with engineered molecules represents a new frontier for therapeutics.

Conventional RFA is a useful treatment option for destruction of hepatic malignancies, both primary and metastatic lesions, as well as a few additional solid cancers [4]. Currently, RFA is limited in the size of the tumors that it can effectively treat [3,5]. Treating tumors larger than 5 cm in diameter with invasive RFA results in incomplete tumor destruction in 10–40% of cases [2,4,5]. The tumor must be treated with an ablation needle precisely placed to assure optimal tumor destruction. In treating hepatic malignancies, the RF energy applied to the invasive needle electrode produces indiscriminate heating of any tissue type within which it is placed, including normal liver parenchyma, bile ducts, and other organs or structures in proximity to the malignant cells. Additionally, tumor location can prevent percutaneous or laparoscopic (minimally invasive) approaches for RFA. Theoretically, an external RF field generator would eliminate the need for an invasive needle electrode, be able to focus energy at any tumor location and body site, and not be limited by the size of the tumor. In order to produce thermally-induced cancer cell death in response to the RF field *in vivo*, intracellular or intratumoral resonant or metallic heat-producing molecules are required. GNPs are excellent conductors of electrical and thermal energy and in our system provide non-specific RF targeting to human gastrointestinal cancer cells *in vitro*. GNPs appear to be taken into the cancer cells *in vitro *by endocytosis with evidence of cytoplasmic vesicles containing GNPs seen in our electron microscopy images. We have evidence that solid tumor treatment *in vivo *is feasible and effective using intracellular single-walled carbon nanotubes as the heating releasing entity [24], but this *in vivo *approach needs to be validated using GNPs. Ideally, GNPs can be targeted to malignant cells *in vivo *by attaching tumor-specific or tumor-related targeting molecules such as antibodies, peptides, or pharmacologic agents.

The data here represent the combination of these two novel approaches, intracellular GNPs and a unique non-invasive RF field generator. Other researchers have demonstrated some decreased cellular proliferation with GNP exposure. For example, GNPs have been shown to have anti-proliferative activity in multiple myeloma cells [25]. GNPs were not cytotoxic to these myeloma cells and the anti-proliferative activity was reversible. GNPs are not cytotoxic or anti-proliferative *in vitro *in the two solid tumor cancer cell lines studied here. This is demonstrated in the MTT assays, PI-FACS control specimens without RF, and the normal TEM appearance of GNP-treated Hep3B and Panc-1 cancer cells not exposed to the RF field. Transmission electron microscopy was also able to confirm the internalization of GNPs into these human gastrointestinal cancer cell lines. As seen in Fig. 2, the GNPs in the untreated cells appear to be within endosomes.

Gold salts have been utilized as an immunomodulator for decades in the United States, but they are not considered cytotoxic [26]. GNPs are particularly interesting as a therapeutic target for non-invasive RF because a number of gold preparations are already used in clinical practice. Intramuscular gold and oral gold compounds are already approved for use by the Food and Drug Administration as a therapeutic agent for rheumatoid arthritis [25,26]. These gold formulations used to treat rheumatoid arthritis are well tolerated in a majority of patients [27]. Parenteral gold typically causes side effects in about 35% of patients, which can include dermatitis, diarrhea, or stomatitis. More severe reactions such as nephritis, bone marrow suppression, colitis, and hepatotoxicity are more rarely observed [28,29]. While the toxicity profile for colloidal gold and GNPs does not demonstrate any hematologic or biochemical sequelae, these gold formulations are not currently used in the treatment of rheumatoid arthritis [30]. Our findings here are consistent with reports describing the current therapeutic use of gold for rheumatoid arthritis with no apparent cytotoxicity to our cell lines *in vitro *[26,31,32]. However, the potential systemic toxicity of GNPs in humans is not currently known and requires further preclinical investigation before these molecules are deemed safe for clinical trials. We believe this approach is promising and have initiated preclinical toxicity studies of GNPs in normal and tumor-bearing animals.

Once the GNPs are internalized, they serve as target molecules to produce increased intracellular heat when exposed to the external RF field. The PI FACS data displayed in Table 1 and Figure 3 demonstrates the increased percentage of cell death in the GNP-treated cells exposed to the external RF field. TEM reveals disruption and destruction of normal intracellular structures and architecture. Importantly, the difference in RF-induced cytotoxicity between the GNP-treated group and control cells is significant, with over 98% cell death in both Panc-1 and Hep3B GNP-treated groups. The cytotoxicity noted in the control cells is related to non-specific ionic stimulation and heat production that is known to occur in powerful RF fields [33]. It will be important to study our system carefully to determine the optimal duration of RF exposure, use of pulsed RF, and RF field strengths necessary to produce lethal injury in GNP-laden malignant cells while avoiding RF-induced damage to normal cells. The current experiments indicate that GNPs are suitable targets for RF-induced thermal destruction of cancer cells. It is possible that shorter duration RF exposures may be sufficient to produce apoptosis-inducing injury in cancer cells bearing GNPs while sparing adjacent normal cells not containing GNPs. To achieve this goal, methods to deliver the GNPs exclusively or preferentially to the cancer cells must be investigated.

It is clear from our data that as an intracellular target molecule, GNPs release substantial heat in the nanoenvironment after exposure to a high-voltage focused RF field. This heating occurs very rapidly (as quickly as one minute) *in vitro*. The amount of heating related to the intracellular GNPs represents a marked difference compared with the ion rich control samples which contain DMEM and 10% fetal calf serum, but no GNPs. The GNPs in the current experimental system are acting as nonspecific target molecules. Future experimental steps include wrapping the surface of GNPs with a targeting agent to selectively deliver GNPs to malignant cells followed by generation of hyperthermia using non-invasive RF. In this respect, the surface area of GNPs is an important factor for surface functionalization. The reason for selecting GNPs as a target for this study is manifold: 1) recently, GNPs have been used in various biomedical applications [13,15,19,22,23,34-42]; 2) as mentioned earlier, colloidal gold and gold compounds have a long history of use in humans [43,44]; 3) they are easy to synthesize and characterize due to the presence of a characteristic surface plasmon resonance (SPR) band (absent in all other organic based nanoparticles systems such as polymeric nanoparticles, liposomal nanoparticles, dendrimeric nanoparticles) [23]; 4) their surface chemistry is relatively simple and surface modification (attaching biomolecules including proteins/antibodies, drugs, and DNA) can be done fairly easily [45-49] than other relevant technologies (liposomal, polymeric, etc); 5) they have high surface area that allows multiple drug loading on a single particle, and most importantly, 6) they are biocompatible and do not elicit toxic effects [22,30,41,50-52]. Recent *in vitro *and *in vivo *reports have confirmed the absence of chronic biochemical and hematological toxicity in mice up to one year after injection of GNPs (1.9 nm in diameter) [30]. All of these qualities associated with GNPs make it a potentially ideal molecule for targeted hyperthermia.

We selected GNP of ~5 nm diameter due to the simple synthesis process and high surface area with this size. A spherical GNP of 5 nm size has 23% surface atoms, whereas a 10 nm particle has 11.5%, a 50 nm particle has 2.3% surface atoms and a 1000 nm particle has only 0.2% surface atoms [53,54]. Due to this higher surface atoms feature, a 5 nm particle will have maximum loading capacity with a minimum gold content. Furthermore, the small size of these nanoparticles may allow them to escape uptake by mononuclear phagocytic cells and penetrate through the smallest capillary pores within the human vasculature.

The preliminary findings here are promising for the use of GNPs as a heat-releasing substrate for this completely non-invasive RF technique. Development of an *in vivo *tumor model will be important to establish this technique as a feasible treatment modality for solid tumors. Additionally, specific targeting of the GNPs, either through antibodies, peptides, or other entities will likely be necessary to provide tumor-only destruction by the RF and thus, provide significant advantage over current invasive radiofrequency technology.

## Conclusion

Our preliminary studies here indicate that GNPs added to the media of human cancer cells *in vitro *are taken up and localized in vesicles in the cytoplasm of the cells. The presence of these GNP-laden vesicles has no apparent cytotoxic or anti-proliferative effect on the cells. Furthermore, GNPs exposed to an external, non-invasive 13.56 MHz RF field release significant amounts of heat, in fact often sufficient to raise water temperatures to the boiling point. Exposing GNP-bearing human cancer cells to this external RF field *in vitro *produced dose-dependent lethal injury in > 96% of the cells. Based on these promising results, we have initiated studies to evaluate *in vitro *cytotoxicities of GNPs and methods to target the GNPs to tumors *in vivo *to affect RF-induced thermal destruction of malignant tumors.

## Methods

### GNP production

GNPs were prepared using previously described methods. In brief, 50 mL of aqueous solution containing 4.3 mg of solid sodium borohydride was added to 100 mL of 100 μmol/L aqueous solution of tetrachloroauric acid under vigorous stirring for at least 12 hours. Nanogold particles formed and were then filtered through a 0.22 μm filter. Transmission electron microscopy (TEM) was utilized to confirm uniform creation of 5 nm GNPs [25].

### External radiofrequency field generator

A variable power 0–2 KW 13.56 MHz RF field generator (Therm Med LLC, Erie, Pennsylvania, USA) was built to specifications for use in these experiments. The RF generator was connected to a high Q coupling system (Therm Med LLC, Erie, Pennsylvania, USA) with a Tx head (focused end-fired antenna circuit) and reciprocal Rx head (as a return for the generator) mounted on a swivel bracket allowing the RF field to be oriented in either a horizontal or vertical direction. The distance between the heads was also adjustable. The coaxial end-fire circuit in the Tx head produced an electronic focused RF field up to 15 cm in diameter. Each time the RF field was activated, the couplers were checked and fine tuned to assure that there was no reflective power between the Rx and Tx heads. The electromagnetic field strength between the Tx and Rx head was established in a Farraday-shielded room to exclude any interference from external RF sources. The field was measured using a Hewlett Packard Spectrum Analyzer (model 8566B, Agilent, Santa Clara, California, USA) and an isotropic field monitor and probe (models FM2004 and FP2000, Amplifier Research Inc., Souderton, Pennsylvania, USA). In our instrument, output powers of 200, 400, 600, 800, and 1000 watts were used, giving maximum estimated electric field strengths (Ep) 2.5 cm from the Tx head of 8.0, 10.1, 12.4, 14.3, and 16.0 kV/m, respectively.

### RF heating of GNPs

Thermal properties of GNPs in the external RF field were obtained using 1.0 mL GNP samples at concentrations of 1.1 μM/L, 11.1 μM/L, 33.5 μM/L, and 67 μM/L in deionized water. The RF field was generated in the horizontal plane at powers of 200 W, 400 W, 600 W, and 800 W with exposure times up to 5 minutes or until boiling of the solution occurred. Temperature measurements were obtained using the FOT Fluoroptic Lab Kit (Luxtron Corp, Santa Clara, California, USA). Samples for each concentration and power were repeated in triplicate at the minimum.

### Human gastrointestinal cancer cell lines

Panc-1 and Hep3B cells were utilized for all experiments (American Type Culture Collection, Bethesda, Maryland, USA). The cells were maintained in standard culture conditions with 10% fetal calf serum and penicillin/streptomycin at 37°C. For experimental purposes, each cell line was only utilized from passages 2–9.

### MTT assay

Hep3B and Panc-1 cells were plated in 96-well plates at a density between 4–8,000 cells per well. Nearly confluent (10–20,000 cells per well) Hep3B and Panc-1 cell lines had increasing concentrations of GNPs in media added (1 μM/L, 10 μM/L, 67 μM/L) with media alone without GNPs as a control. Cells were maintained at 37°C for 24 hours after adding GNPs. 3-(4,5-Dimethylthiazol2-yl)-2.5-diphenyltetrazolium bromide (MTT) was then added to each well and incubated for 4 hours. Absorbance was interpreted at 570 nm for each well. Each concentration was repeated in triplicate with five wells in each group for a total of 15 samples per tray per condition. MTT assay could not be combined with RF treatment. The 96-well plates were too large to reliably focus the RF field on a single GNP concentration in a uniform fashion.

### External RF treatment

Hep3B and Panc-1 cells were grown to near confluence on 60 mm Pyrex dishes. Cells were incubated for 24 hours in media with 1, 10, or 67 μM/L GNPs, or with media alone. Media containing GNPs not taken up by the cancer cells was aspirated and fresh media without GNPs was then added to each dish. The cell cultures were then treated with RF exposure times of 1, 2 or 5 minutes. Culture temperatures were measured prior to and at completion of RF exposure with a FOT Fluoroptic Lab Kit (Luxtron Corp, Santa Clara, California, USA). Cells were then returned to the 37°C incubator for 18 hours. All RF exposure times were repeated in triplicate at the minimum.

### Propidium iodide-fluorescent activated cell sorting (PI-FACS)

Cells were harvested after completion of the post-RF incubation and fixed in 95% EtOH. Cells were prepped with propidium iodide (PI) and DNase free RNase. The BD FACS Calibur (BD, San Jose, California, USA) was utilized as the fluorescence activated cell sorter (FACS). CellQuestPro (BD, San Jose, California, USA) analyzed the data.

### Transmission electron microscopy

Cells were harvested in similar fashion to FACS protocol following RF exposure as well as control conditions. The cells were then fixed in 10% formalin. Cells were rinsed for 30 minutes in 3 changes of 0.1 M phosphate buffer, pH 7.2, followed by a 1 hour postfix in phosphate-buffered 1% OsO4. After washing with distilled water thrice for 30 minutes, the tissue was en-bloc stained with 2% uranyl acetate for 30 minutes at 60°C. The cells were then rinsed again in three changes of distilled water, dehydrated in progressively higher concentrations of ethanol and 100% propylene oxide, and embedded in Spurr's resin. Thin (90 nm) sections were cut on a Reichert Ultracut E ultramicrotome, placed on 200 mesh copper grids, and stained with lead citrate. Micrographs were taken on a TECNAI 12 (FEI/Philips, Hillsboro, Oregon, USA) operating at 120 KV.

## Competing interests

The author(s) declare that they have no competing interests.

## Authors' contributions

CJG participated in the conception of these studies, performed cell studies and established heating rates of GNPs in solution, analyzed data and wrote the manuscript. CP performed TEM of cancer cells receiving GNPs. RB performed TEM of cancer cells receiving GNPs. PM provided GNPs, participated in the conception of these studies, and participated in writing the manuscript. SAC conceived experimental design and studies, analyzed data, and participated in writing this manuscript.

All authors read and approved the final manuscript
